# Unlocking the Healing Potential: A Comprehensive Review of Ecology and Biology of Medical‐Grade Honey in Wound Management and Tissue Regeneration

**DOI:** 10.1002/hsr2.70240

**Published:** 2025-01-16

**Authors:** Parmis Barazesh, Helia Hajihassani, Fatemeh Motalebi, Seyedeh Mobina Hosseini Neiresi, Romina Hajihassani, Ahmad Reza Mehrabian

**Affiliations:** ^1^ Faculty of Life Sciences and Biotechnology Shahid Beheshti University Tehran Iran; ^2^ Faculty of Biological Sciences University of Tehran Tehran Iran; ^3^ Bee Products Research Centre Shahid Beheshti University Tehran Iran

**Keywords:** antibacterial properties, innovative applications, medical‐grade honey, wound healing

## Abstract

**Background and Aims:**

Honey has long been studied for its healing abilities in wound care. This narrative review examines its properties and their impact on wound healing, particularly its ability to accelerate wound closure and promote tissue regeneration. The review focuses on how honey's botanical origins affect its medical properties and wound‐healing capabilities. Finally, clinical studies on honey's effectiveness in wound healing were reviewed compared to traditional treatments.

**Methods:**

Relevant keywords were searched in databases, yielding 1250 documents. After excluding nonrelevant sources, 450 documents were refined, and 167 articles were selected based on thematic alignment and originality. Data extraction focused on study design, intervention details, and outcomes, with quality assessed using standardized criteria. The study adhered to CONSORT and SANRA guidelines to ensure methodological rigor and reporting transparency.

**Results:**

Honey‐based medical products have demonstrated significant antibacterial, anti‐inflammatory, and tissue‐regenerative properties, making them highly effective in improving wound healing outcomes, particularly in chronic and burn wounds. These products have also been shown to reduce infection rates and hospital stays. While some studies have reported positive outcomes in accelerating the healing process, others have found no significant difference compared to conventional treatments.

**Conclusion:**

Medical‐grade honey (MGH) holds potential for wound care due to its versatility, though variations in its composition present challenges. Further research is needed to optimize its clinical use. The effectiveness of MGH in wound healing remains debated, with mixed results from trials. Genetic modification of bees to enhance MGH's properties could make it more competitive against conventional treatments. Honey‐based medications could reduce costs, improve energy efficiency, and have minimal side effects. Rigorous research is necessary to determine optimal use and fully unlock MGH's potential, which could revolutionize wound management globally.

## 1 Introduction

### 1.1 Honey in the Medical World

Honey, a natural sweetener cherished for its delectable taste, holds a rich history spanning approximately 8000 years, evidenced by Stone Age depictions [1, 2, 3]. Beyond its culinary appeal, honey has long been revered for its medicinal properties, transcending cultures and civilizations [4, 5]. Ancient societies such as the Egyptians, Assyrians, Greeks, and Romans utilized honey, often combined with herbs and essential oils, to treat various ailments ranging from burns to gastrointestinal disorders [6, 7, 8]. Despite falling out of favor with the advent of antibiotics in the 1940s, honey has recently experienced a resurgence in clinical medicine [9]. This revival is spurred by the escalating threat of antibiotic‐resistant bacteria and compelling evidence, both in vitro and in vivo, showcasing honey's efficacy as a natural wound treatment and broad‐spectrum antibacterial agent [10, 11, 12]. Rich in anti‐inflammatory, antioxidant, and antimicrobial compounds, honey is a testament to nature's healing prowess [13]. From inhibiting oxidative processes to bolstering the immune system, honey's multifaceted benefits extend beyond its delightful taste [14, 15]. As we delve into its intricate biochemistry and historical significance, we uncover a timeless remedy poised to shape the future of modern medicine [16].

**Figure 1:**
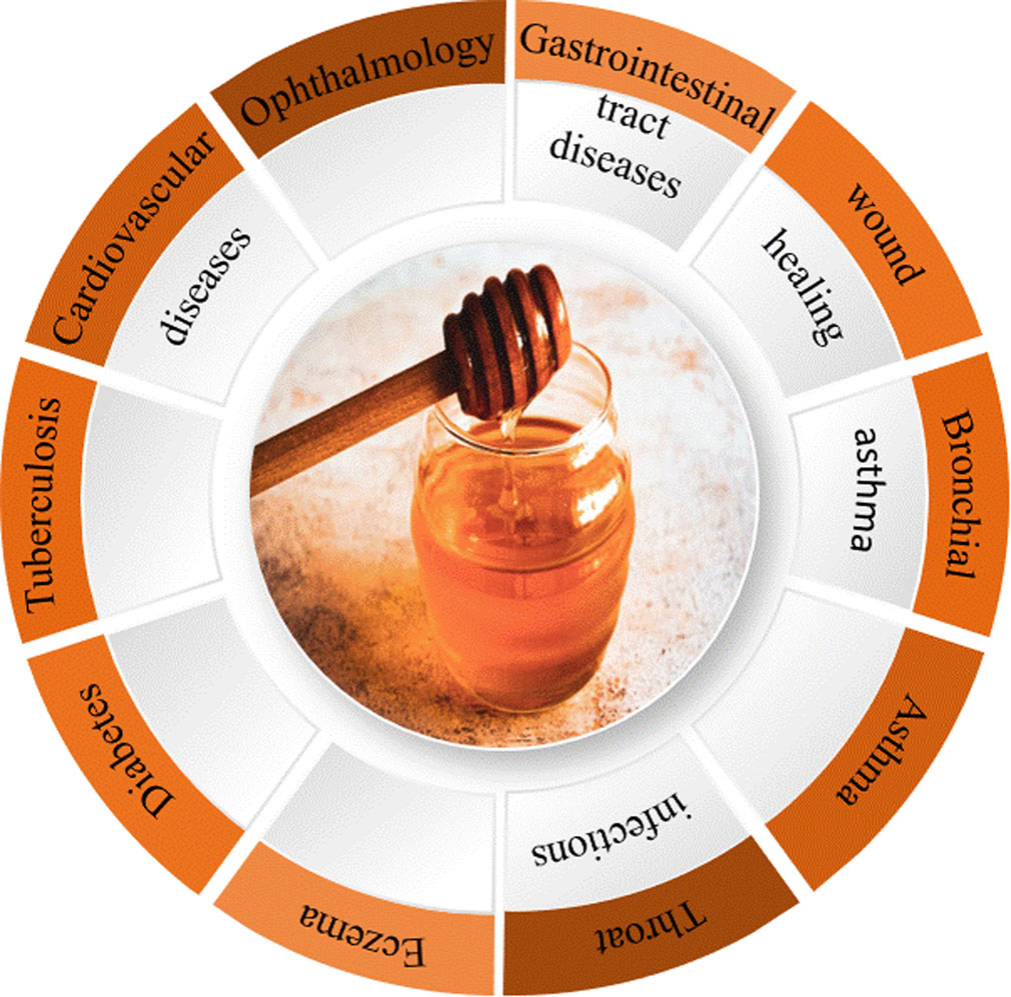
Medical application of honey.

Honey is crucial to human health, whether ingested orally or applied topically [17, 18]. Fir honey and sunflower honey have been proven to help with respiratory disorders by fluidizing bronchial secretions, and mountain honey offers potential advantages in allergies and pulmonary diseases [19]. Figure 1 illustrates various applications of honey in the medical world.

While honey may have general health benefits, such as enhancing immune function or providing antioxidants, these systemic effects do not directly contribute to wound healing. The topical application of medical‐grade honey(MGH) allows the active components, like methylglyoxal (MGO) and H₂O₂, to work directly on the wound surface, providing localized antimicrobial action and promoting faster wound closure [20, 21].

### 1.2 Role of Sterilizing of MGH

The final MGH product must undergo sterilization to ensure it is devoid of bacterial endospores, including Clostridium botulinum, Clostridium tetani, and other potentially harmful microorganisms. This is essential to mitigate the risk of botulism and other associated morbidities [22].

However, sterilization techniques cannot remove other potential contaminants like pesticides or heavy metals. To properly remove these pollutants, different purification techniques such as filtration, adsorption, or chemical treatments are required [23, 24].

Enzymes, vitamins, and phenolic compounds are among the essential components of honey that may be destroyed by thermal sterilization at high temperatures. Additionally, this process may lead to the formation of 5‐hydroxymethylfurfural (HMF), a marker indicative of honey quality deterioration [25, 26].

Bee venom contains a variety of proteins and peptides, such as melittin, phospholipase A2, and hyaluronidase. Sterilization techniques cannot neutralize these components. Instead, these proteins and peptides require purification processes like filtration, adsorption, or chemical treatment to be effectively neutralized [26, 27].

Gamma irradiation is the standard method for sterilizing medical devices and food products to guarantee honey's safe use and consumption. Gamma radiation at a dose of 10 kGy can decrease the moisture content and change the odor of honey [28]. However, at this dose, the decrease in the defensin‐1 content did not affect the antibacterial activity of irradiated honey against Gram‐positive bacteria [29]. In a study, no significant change was found in antibacterial activity even when the radiation was increased to 50 kGy [30].

### 1.3 Mechanisms of MGH in Wound Healing

A wound is any disruption of the skin and underlying tissues, which can be classified into several types. Cuts, abrasions, and surgical incisions are examples of acute wounds, which often heal without problems in the anticipated amount of time. Chronic wounds, including diabetic ulcers, pressure ulcers, and venous leg ulcers, require a longer time to heal because of underlying medical conditions such as low blood flow or infections. Burns can range from first‐degree to third‐degree, depending on whether they arise from thermal, chemical, or electrical sources. Wounds that bacteria or other pathogens have infected are considered infected wounds, as they may cause delayed healing or even systemic illness [31, 32].

Honey is included in the National Health Service NHS protocols for wound management, underscoring its recognized therapeutic benefits [33]. Its broad‐ranging effects on wound healing have been demonstrated, and it plays a significant role in this process. Wound healing is a multifaceted process involving various factors such as growth factors, cells, proteinases, and extracellular matrix constituents [34, 35, 36, 37]. It comprises four interconnected phases: hemostasis, inflammation, proliferation, and tissue remodeling or resolution [38]. Hemostasis initiates blood clotting and exudate formation to stop bleeding [39]. The subsequent inflammatory phase involves clearing debris and preventing microbial invasion [40].

**Figure 2:**
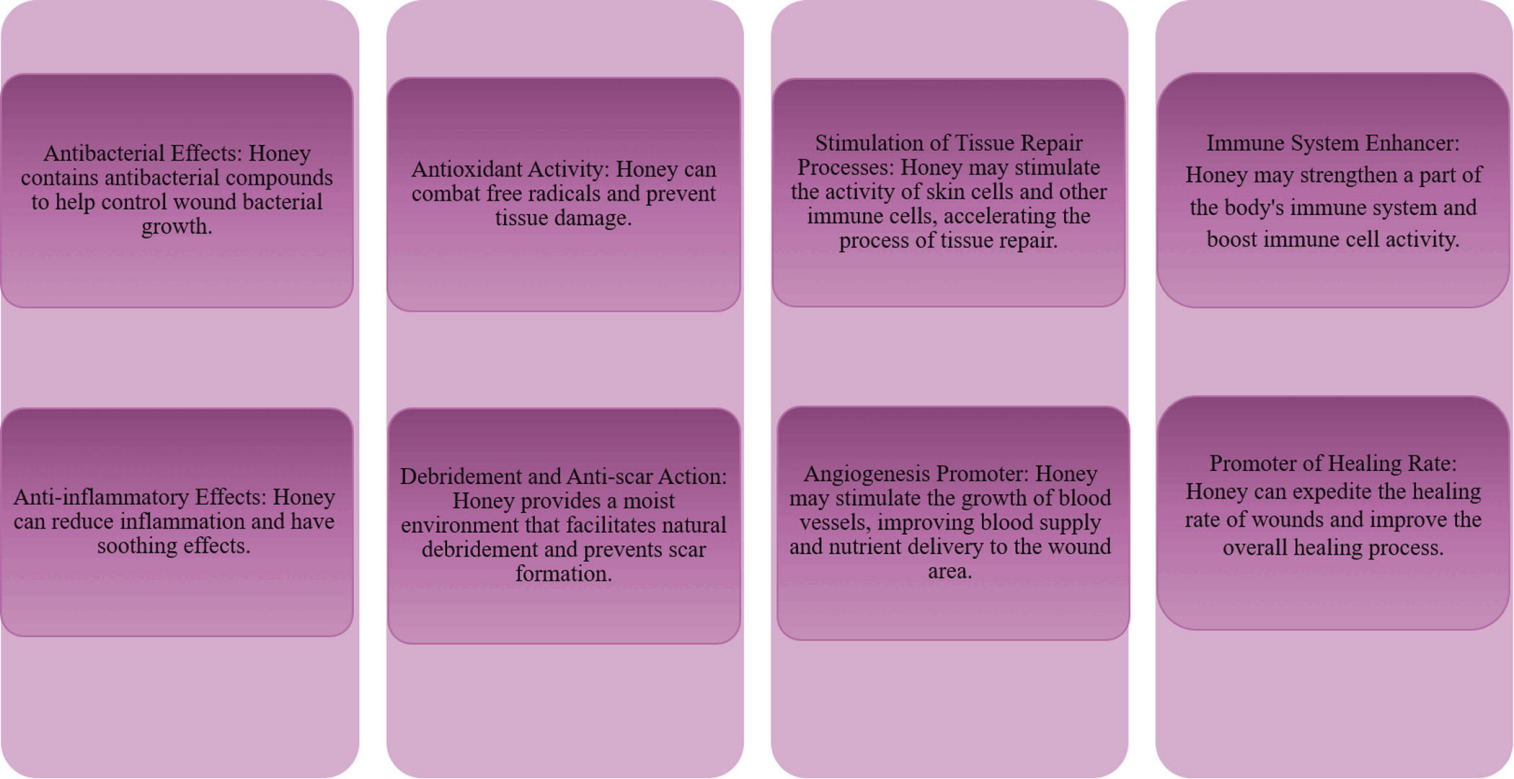
Mechanisms of MGH in wound healing.

Macrophages release cytokines and growth factors to promote angiogenesis and attract essential cells like keratinocytes, fibroblasts, and endothelial cells [41]. During proliferation, epithelialization occurs, forming granulation tissue [42]. Factors affecting wound healing can be local or systemic. Local factors influence physical wound characteristics, while systemic factors impact overall health and recovery [43]. The mechanisms of MGH in wound healing are illustrated in Figure 2.

Throughout each stage of wound healing, honey exhibits distinct effects. It possesses antimicrobial properties, modulates pH, enhances antioxidant activity, stimulates peroxide production, and releases various cytokines during inflammation [44]. In the proliferative phase, honey reduces edema and exudate while promoting epithelialization and granulation [45]. It aids in wound remodeling and prevents scarring. Honey also encourages the activity of certain enzymes and growth factors, contributing to the healing process [46, 47].

Honey is an effective wound‐healing agent due to its cost‐effectiveness, nontoxic nature, and ability to moisturize wounds without adhering to them. It aids in healing, including autolytic debridement, angiogenesis, and granulation. Rich in nutrients and bioactive compounds, honey exhibits antioxidant properties and supports immune function [48, 49, 50]. Thyme honey has shown promise in preventing or treating oral mucositis in cancer patients. Despite its benefits, caution is necessary regarding honey quality and application, particularly in chronic wound healing [51, 52].

**Figure 3:**
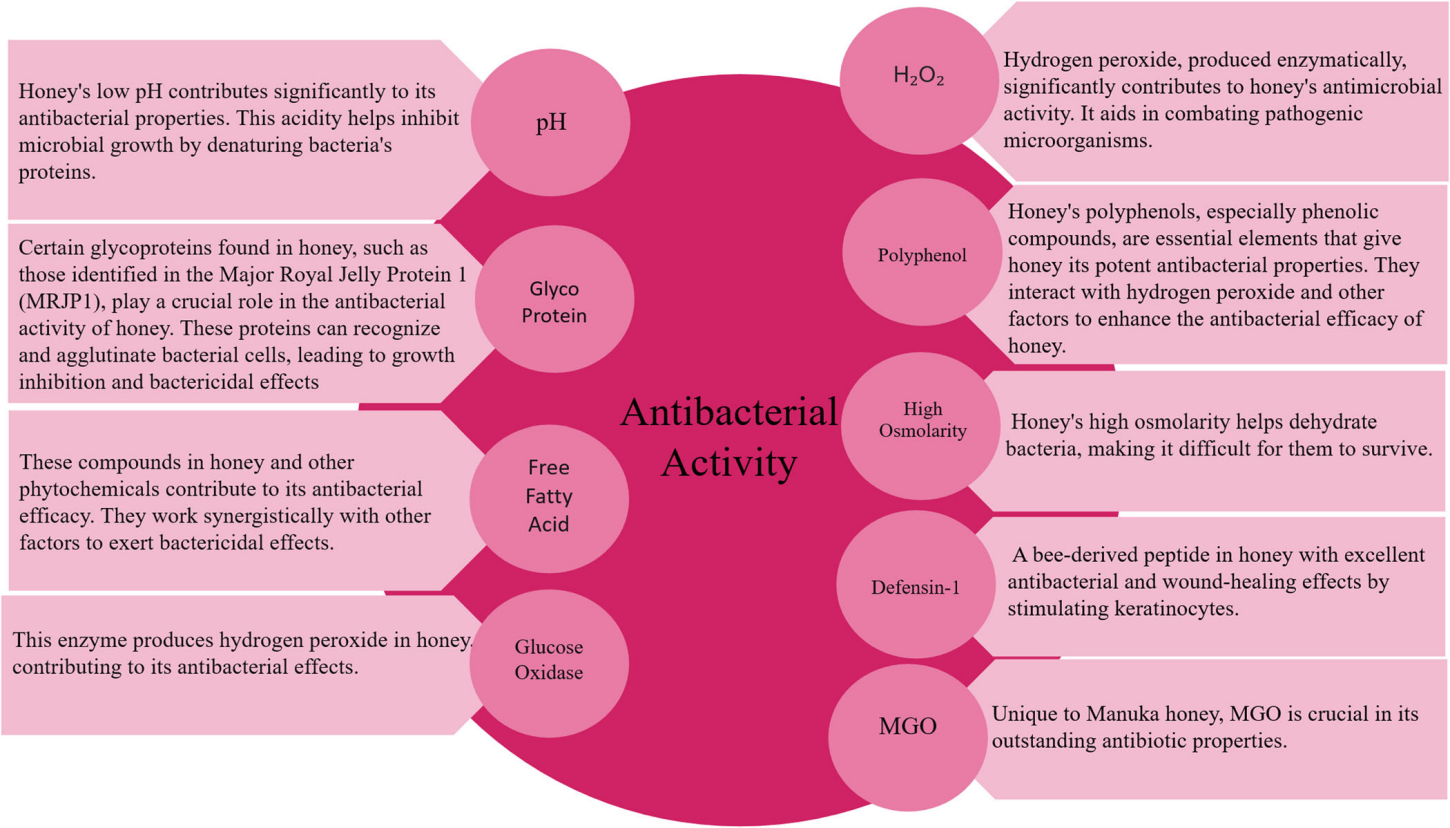
Factors contributing to the antibacterial effects of honey [18, 53, 54, 55, 56, 57, 58, 59].

One of the critical mechanisms of MGH in wound healing is its antibacterial activity. MGH exerts its antibacterial effects by disrupting biofilm, making bacteria more susceptible to treatment. It promotes an environment that prevents bacterial proliferation, enhancing overall wound healing [53]. Figure 3 illustrates the factors involved in honey's antibacterial activity.

Among the various MGH available, Manuka honey, derived from the nectar of the *Leptospermum scoparium* tree native to New Zealand and Australia, employs unique mechanisms in wound care [60]. MGO is a compound that forms in Manuka honey from dihydroxyacetone (DHA) and is found in the nectar of Manuka flowers. MGO exerts antibacterial effects by modifying bacterial proteins through glycation. This process binds MGO to amino groups on proteins, causing them to lose function and leading to bacterial cell death. Additionally, MGO disrupts bacterial cellular functions by interfering with DNA replication and cell division, inhibiting bacterial proliferation [61, 62].

The low pH of Manuka honey enhances the effectiveness of MGO. Its high osmolarity helps preserve H₂O₂. This combination creates a comprehensive approach to bacterial inhibition. As a result, Manuka honey is particularly effective in treating wounds infected with antibiotic‐resistant bacteria like *Methicillin‐resistant Staphylococcus aureus* (MRSA) [63].

Some other MGH exhibit significant antibacterial potency through mechanisms similar to Manuka honey. Revamil honey shows rapid bactericidal effects against *Bacillus subtilis*, *Escherichia coli*, and *Pseudomonas aeruginosa*, primarily due to bee defensin‐1 and H₂O₂. Gelam honey also demonstrates antibacterial activity against pathogens such as *Staphylococcus aureus* and *E. coli* through both peroxide and non‐peroxide mechanisms [64, 65, 66].

### 1.4 Phytochemical Components of Honey and Biological Effects

Numerous essential phytochemicals, such as flavonoids and phenolic compounds, make honey a solid antimicrobial. These compounds mostly come from the plants of their geographical origin. These factors are used to certify and authenticate the honey; thus, a wide range of physicochemical characteristics is seen, including moisture, pH, electrical conductivity, organic acids, etc [67].

Phenolic acids and flavonoids are secondary metabolites of herbs and are among the most essential compounds in honey. The phenols in honey are collected from nectar, honeydew, propolis, or pollen and function as antioxidants. Interestingly, the color of honey correlates with its antioxidant potential [68].

Polyphenols, as the name suggests, consist of many phenol units. They are either flavonoids or non‐flavonoids [69]. Honey contains various types of the mentioned phytochemical groups, and even though they are not significant in quantity, they carry out most of honey's therapeutic features [70]. Some factors specify different subclasses of these compounds, including the number of phenol units they comprise, the substituent groups, and the type of connection between the units in a molecule [71].

A flavonoid is a 15‐carbon backbone accompanied by two phenyl groups and a heterocyclic unit. Flavonoids include many groups: flavones, flavanols, flavanones, and dihydroflavonols. Non‐flavonoids contain phenolic acids, lignans, hydrolyzable tannins, coumarins, and condensed tannins. Some examples of them would be cinnamic acids and their esters. These are all known as proteinaceous, nonperoxide molecules, in addition to lysozyme, bee peptides, and MGO [71, 72, 73, 74, 75]. Table 1 illustrates common phenolic compounds in honey and their mechanisms in wound healing.

**Table 1:** Common phenolic compounds in honey and its mechanisms in wound healing.

Common phenolic compounds and their structures	Botanical origins	Mechanisms in wound healing	References
Apigenin 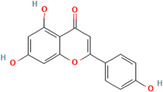	Acacia (Robinia pseudo acacia) Milkweed (*Asclepias syriaca*) Linden (Tilia spp.) Goldenrod (*Solidago gigantea*) Buckwheat (*Fagopyrum esculentum* Moench) Honeydew Rapeseed (*Brassica napus*) Gelam (*Melaleuca cajuputi* powell)	M2‐type macrophages↑, expression of miR‐21↑ Apigenin's effects on macrophage polarization and wound healing are mediated through the TLR4/Myd88/NF‐κB signaling pathway.	[76, 77, 78, 79, 80]
Caffeic acid 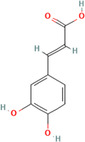	Acacia (Robinia pseudo acacia) Milkweed (*Asclepias syriaca*) Linden (Tilia spp.) Goldenrod (*Solidago gigantea*) Eucalyptus (Eucalyptus) Manuka (*Leptospermum scoparium*) Sunflower (Helianthus) Lavender (Lavandula) Orange (*Citrus sinensis*) Buckwheat (*Fagopyrum esculentum* Moench) Chestnut (*Castanea sativa*) Gelam (*Melaleuca cajuputi* powell)	Glutathione levels↑ Malondialdehyde↓ Levels Vascular endothelial growth factor VEGF↑	[76, 78, 81, 82, 83, 84, 85]
Chlorogenic acid 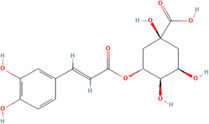	Milkweed (*Asclepias syriaca*) Linden (Tilia spp.) Honeydew honeys Buckwheat (*Fagopyrum esculentum* Moench) Gelam (*Melaleuca cajuputi* powell)	It helps to control inflammation, accelerate collagen synthesis and promote fibroblast proliferation.	[76, 84, 86, 87]
Ferulic acid 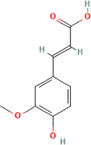	Acacia (*Robinia pseudoacacia*) Milkweed (*Asclepias syriaca*) Linden (Tilia spp.) Goldenrod (*Solidago gigantea*) Eucalyptus (Eucalyptus) Manuka (*Leptospermum scoparium*) Sunflower (Helianthus) Lavender (Lavandula) Orange (*Citrus sinensis*) Rosemary (Salvia rosmarinus) Lime (*citrus aurantiifolia*) Rapeseed (*Brassica napus*) Raspberry (*Rubus idaeus*) Buckwheat (*Fagopyrum esculentum* Moench) Honeydew Gelam (*Melaleuca cajuputi* powell)	Enhance angiogenesis, antioxidant enzymes↑	[76, 78, 81, 84, 88, 89]
Galangin 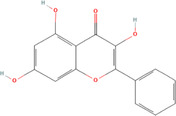	Acacia (*Robinia pseudoacacia*) Milkweed (*Asclepias syriaca*) Linden (Tilia spp.) Goldenrod (*Solidago gigantea*) Manuka (*Leptospermum scoparium*) Sunflower (Helianthus)	Modulates TGFβ–SMAD signal, expressions of type I collagen, type III collagen, and TGF‐β1↓, Expression of Smad7↑	[76, 81, 90, 91]
Gentisic acid 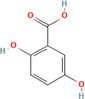	Milkweed (*Asclepias syriaca*) Linden (Tilia spp.) Pine (Pinus)	Acidity↑, Proteases activity↓	[76, 92, 93]
*p*‐coumaric acid 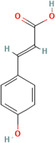	Acacia (*Robinia pseudoacacia*) Milkweed (*Asclepias syriaca*) Linden (Tilia spp.) Goldenrod (*Solidago gigantea*) Lavender (Lavandula) Eucalyptus (Eucalyptus) Sunflower (Helianthus) Buckwheat (*Fagopyrum esculentum* Moench) Peppermint (*Mentha piperita*) Rapeseed (*Brassica napus*) Honeydew Milkvetch (Astragalus) Gelam (*Melaleuca cajuputi* powell) Malaysian Tualang (Koompassia excelsa)	Stimulate collagen synthesis, Promote angiogenesis	[76, 78, 79, 81, 84, 94, 95, 96, 97, 98]
Syringic acid 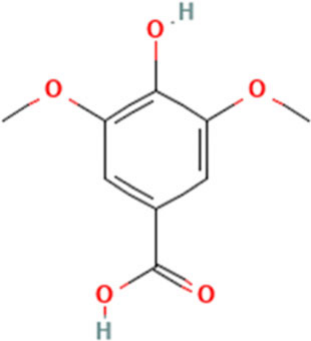	Acacia (*Robinia pseudoacacia*) Milkweed (*Asclepias syriaca*) Linden (Tilia spp.) Goldenrod (*Solidago gigantea*) Honeydew Rapeseed (*Brassica napus*) Buckwheat (*Fagopyrum esculentum* Moench) Manuka (*Leptospermum scoparium*) Malaysian Tualang (Koompassia excelsa) Sunflower (Helianthus) Eucalyptus (Eucalyptus) Lavender (Lavandula)	Cell membrane dysfunction, Enhance angiogenesis↑ Collagen Synthesis↑	[76, 78, 79, 81, 99, 100, 101, 102]
Luteolin 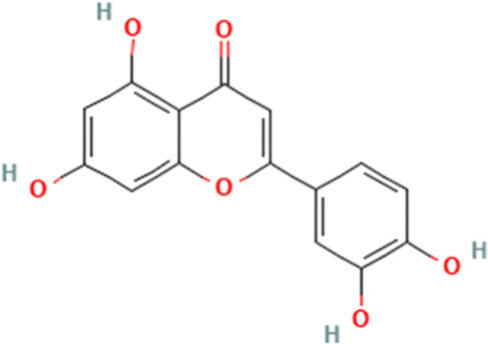	Gelam (*Melaleuca cajuputi* powell) Eucalyptus (Eucalyptus) Lavender (Lavandula) Acacia (*Robinia pseudoacacia*) Manuka (*Leptospermum scoparium*) Heather (Erica) Calluna (Calluna) Rape (Brassica) Sunflower (Helianthus) Rhododendron (Rhododendron) Lime (*citrus aurantiifolia*) Yapunyah (Eucalyptus ochlophobia)	Downregulates inflammatory factors such as matrix metalloproteinase (MMP)‐9, tumor necrosis factor (TNF)‐α, interleukin (IL)‐6, and IL‐1β, inhibit nuclear factor (NF)‐κB pathway	[79, 81, 103, 104, 105]
Gallic acid 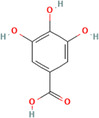	Eucalyptus (Eucalyptus) Manuka (*Leptospermum scoparium*) Sunflower (Helianthus) Lavender (Lavandula) Orange (*Citrus sinensis*) Acacia (*Robinia pseudoacacia*) French Lavender (*Lavandula stoechas*) Malaysian Tualang (Koompassia excelsa) Gelam (*Melaleuca cajuputi* powell) Ulmo (*Eucryphia cordifolia*) Tea tree (Melaleuca alternifolia) Nigella (Nigella) Litchi (*Litchi chinensis*)	It activates factors known to be hallmarks of wound healing, including focal adhesion kinases (FAK), c‐Jun N‐terminal kinases (JNK), and extracellular signal‐regulated kinases (Erk)	[81, 101, 106, 107, 108, 109, 110, 111, 112]

Most honey contains various phenolic acids, such as caffeic acid, coumaric acid, syringic acid, gallic acid, cinnamic acid, ellagic acid, and many others [81, 113]. However, these components make honey richer in rural regions than in urban areas. The difference is known to rise from where the bee colonies are located, not the floral source of the honey [114]. For instance, honey produced by bee colonies near wildflower meadows, forests, and agricultural fields in rural areas frequently exhibits a higher phenolic content, attributed to the diverse and abundant flora in these environments. Consequently, rural honey is often recommended for medical purposes due to its superior phenolic composition and the associated health benefits it confers [115].

### 1.5 The Regeneration Role of Honey

Honey's anti‐inflammatory benefits come from its ability to reduce the production of pro‐inflammatory cytokines and other inflammatory agents. By decreasing the activity of inflammatory transcription factors, which are proteins that help regulate the expression of genes involved in the inflammatory response, like NF‐κB and MAPK, honey helps control wound inflammation [47, 116].

Honey has been shown to stimulate immune system mediators, such as B‐lymphocytes, T‐lymphocytes, and neutrophils, promoting an immune response to infection. By enhancing the immune response, honey helps prevent infections, promotes faster healing, and supports overall immune health [117]. It can also accelerate the regeneration of new tissue by creating an acidic wound environment that favors macrophage action, limits bacterial growth, stimulates cell growth, and prepares development. Honey has been incorporated into tissue‐engineered scaffolds to enhance wound treatment. Various scaffold fabrication techniques are being researched to improve the delivery of honey compounds during wound treatment [118, 119, 120].

### 1.6 Botanical Origin of MGH

Honey's composition and properties vary significantly based on its botanical origin, bee secretions, and geographic location. While different types of honey share similar physicochemical properties, each sample is unique unless harvested simultaneously from the same hive. Honey's phytochemical content and botanical origin influence its antibacterial and anti‐biofilm potentials, critical factors in its wound healing abilities [121, 122].

A 2015 study involving 37 honey samples from 14 botanical sources highlighted the importance of honey's botanical origins in determining its antimicrobial activity. Expert beekeepers identified the origins of these samples and compared them to Manuka honey, emphasizing the strong correlation between antibacterial capabilities and specific physical‐chemical characteristics linked to botanical sources [69, 123].

When selecting honey for treating infected wounds, it is crucial to consider the susceptibility of different bacterial species to honey from various botanical origins. Understanding the relationship between honey's botanical source and antimicrobial activity can guide healthcare providers in choosing the most effective type of honey for wound care based on specific bacterial strains.

### 1.7 Honey‐Based Formulated Medical Products on Wound Healing

Honey has a long history of being used for wound treatment, impacting the natural wound‐healing process by reducing edema and exudation and supporting the stages of hemostasis, inflammation, proliferation, and remodeling [124, 125]. While direct application of honey can pose challenges due to stickiness and leakage issues, incorporating honey into various formulations like hydrogels, dressings, ointments, and pastes has emerged as a solution [126, 127].

Tissue Regeneration Templates have integrated honey into biomaterial tissue templates like Electro spun Templates, Cryogels, and Hydrogels to enhance tissue regeneration by reducing inflammation, combating infections, and promoting tissue integration [128, 129].

Hydrogels combined with honey offer significant advantages as wound dressings that accelerate healing. Advanced Dressing Innovations have led to the development of dressing products containing honey in non‐running gels for efficient wound care [130]. Comparative studies on natural wound treatment substances have highlighted the superior antimicrobial activity of specific formulations like l‐Mesitran soft against *P. aeruginosa* biofilms [131].

Research Findings have shown the effectiveness of nanocomposite hydrogels containing honey in promoting wound healing [132]. Nanofibrous composite membranes containing curcumin and stingless bee honey have notably improved wound healing mechanisms *in vivo and in vitro* experiments [133, 134]. Hydrogel films infused with honey and chitosan have also demonstrated significant efficacy in wound treatment [135]. Furthermore, research studies indicate that alginate hydrogels combined with honey accelerate wound healing [136].

Combining honey with biopolymers like alginate, chitosan, *aloe vera*, and pectin has enhanced antibacterial, anti‐inflammatory, and stimulatory effects for wound care [137, 138]. Bee Product Ointments containing propolis, honey, and apilarnil have shown healing and re‐epithelialization effects on experimental models [139].

### 1.8 In Vitro, In Vivo, and Clinical Trials of MGH

Healthcare providers often encounter challenges when treating wounds, especially chronic ones. Honey, known for its antibacterial properties, has been a traditional remedy for wound care due to its phytochemical composition [140]. Honey's unique properties make it highly effective in treating various types of wounds, particularly chronic wounds, burn wounds, and surgical wounds [141].

According to the results of a systematic review up to 2014, due to the low quality of evidence, it could not be conclusively determined that honey is superior to conventional treatments for many types of wounds, except for partial‐thickness burns [142]. However, some studies have emphasized that honey has enhanced the healing of certain wounds examined in those studies [143, 144].

Manuka honey, with its unique antibacterial activity from compounds like MGO and bee defensin‐1, has been studied in wound‐healing cases such as diabetic foot ulcers and burn injuries [145]. Clinical trials have demonstrated the efficacy of Manuka honey in reducing treatment time and improving outcomes compared to conventional methods [146, 147, 148, 149].

Studies have highlighted the benefits of honey‐impregnated dressings in reducing antibiotic requirements and hospital stays [150]. Honey treatment has shown significant advantages in diabetic foot ulcer management, leading to faster infection clearance, shorter hospital stays, and higher healing rates [151, 152, 153, 154]. Research on different types of honey, such as Jamun honey, has demonstrated notable wound‐healing properties in diabetic models. Studies comparing honey dressing to conventional treatments like povidone‐iodine have shown faster healing times with honey‐based therapies [155, 156, 157].

In various clinical trials, honey has proven effective in reducing infection rates, promoting re‐epithelialization, and improving wound healing outcomes compared to standard treatments like silver sulfadiazine dressing [158]. Honey has also shown positive effects on pain reduction, swelling, granulation tissue formation, and esthetic improvement of surgical wounds [159, 160].

Promising recent outcomes have been reported regarding the improvement of wound healing using MGH, especially in elderly patients who often face difficulties due to comorbidities and age‐related alterations in skin integrity [161, 162].

Honey is a heterogeneous substance containing bee‐derived proteins and pollen, which can potentially cause allergies. In the general population, the prevalence of allergies to honey is less than 0.001%. However, the symptoms of these allergies can vary, ranging from mild allergic reactions to severe systemic responses [163]. Although most skin types can tolerate honey without problems, individuals allergic to bee products should be cautious. Bee venom can cause serious health risks and even lead to severe illness when it stings a person who has a bee allergy. While bee products could benefit some people, using them should be done cautiously. It is essential to ascertain whether the person has any allergies or sensitivities to compounds associated with bees before using bee‐related substances [164, 165].

Overall, honey‐based medical products have shown promising results in preclinical and clinical trials for wound management. Their antibacterial, anti‐inflammatory, and tissue‐regenerative properties make them valuable assets in modern wound care practices. Although certain recent studies have indicated faster healing, other research has not demonstrated a significant advantage over traditional treatments. Table 2 illustrates recent clinical studies investigating the impact of different kinds of honey on human wound healing.

**Table 2:** Recent clinical studies investigating the impact of different kinds of honey on human wound Healing.

Honey name	Wound type	Cases	Outcomes	References
Kelulult	Diabetic	*n* = 30 (gel treatment) *n* = 32 (honey treatment)	The mean percentage reduction in wound size was 45% (SD = 10%) in the honey group compared to 40% (SD = 12%) in the Intrasite gel dressing group. An independent t‐test was conducted to compare the mean reduction between the two groups, with the difference found to be statistically nonsignificant (*p*‐value the nearest hundredth). Both the honey and Intrasite gel groups demonstrated an increase in the median percentage of wound granulation over time; however, no statistically significant interaction effect between treatment type (honey vs. Intrasite gel) and time on wound granulation was observed (*p*‐value the nearest hundredth).	[166]
l‐Mesitran	Venous leg ulcers	*n* = 9 with a total of 11 venous leg ulcers	Treatment with MGH effectively eliminated clinical signs of infection in an average of 2.2 weeks (range: 1–4 weeks). The wounds were fully healed after an average of 7 weeks of treatment.	[162]
l‐Mesitran (ointment, Tulle and foam)	Diabetic foot ulcer	*n* = 5	A reduction in wound area was observed within the first 40 days of treatment with MGH, as measured using planimetry, a precise method for measuring wound dimensions. Additionally, the study monitored patients' blood glucose levels and found no significant increase in glycated hemoglobin (HbA1c) or glycaemia levels throughout the treatment period.	[167]
l‐Mesitran Soft	Cold sores	*n* = 29	The average healing time for cold sores was found to be 5.8 days with l‐Mesitran (medical‐grade honey) compared to 10.0 days with conventional treatments. A paired t‐test was conducted to compare the mean healing times between the two treatment groups. The analysis demonstrated a statistically significant reduction in healing time with l‐Mesitran, with a P‐value of less than 0.0001 (*p* < .0001), indicating that medical‐grade honey was more effective than conventional treatments in accelerating cold sore recovery.	[144]

## 2 Materials and Methods

This review offers a detailed approach for identifying, selecting, and evaluating pertinent scientific literature on a particular topic or issue. The data extraction process is largely influenced by factors such as the hypothesis, research question, study necessity, the nature of the data being analyzed, and the estimated volume of available data. Crafting an appropriate, precise, and comprehensive search strategy is essential for conducting an effective review.

An initial review was conducted to identify relevant keywords, including “Medical‐Grade Honey,” “Wound Healing,” “Antibacterial Properties,” and “Innovative Applications.” Pertinent databases such as PubMed, Science Direct, Google Scholar, and Springer were meticulously selected and searched. The search process involved querying article titles and keywords within these databases until matches were found with those employed in previous studies.

The extensive search yielded 1250 documents. Books, reviews, reports, and conference papers were excluded to refine the data set. Additionally, sources lacking full‐text accessibility were omitted, resulting in a refined selection of 450 documents. A comprehensive evaluation based on relevance criteria was then conducted to determine the inclusion of documents in the research. This evaluation considered factors such as thematic alignment with research variables, originality of research sources, and overall relevance.

Findings from recent randomized controlled trials and case reports were collected and compared with prior reviews and experimental studies. These datasets, as well as those from comparative studies, were cohesively integrated into the article. Information regarding the study design was sourced from the methods, results, and discussion sections of the articles.

The outcomes were focused on key healing metrics, such as wound size reduction and healing times, along with the collection of quantitative data and statistical results to objectively assess treatment efficacy. Details related to the interventions, including the type of honey used, the application process, and the duration of treatment, were documented directly from the methods sections.

Accordingly, to ensure reliability and validity, the quality of the included studies was assessed using standardized criteria. The standardized criteria used for assessing the quality of the included studies were based on the Scale for the Assessment of Narrative Review Articles (SANRA) [168].

The refined selection identified 167 articles and research theses as suitable for inclusion in the review. The sources were grouped into three main categories using Microsoft Excel (version 2019):

1. Properties of MGH in wound healing, including its mechanisms and effects.
2. Botanical origin of honey.
3. In vitro, in vivo, and clinical studies conducted in this field.

The manuscript was subsequently organized and composed following these categorizations. While this review only reviews RCTs conducted, adherence to the relevant principles of the CONSORT guidelines ensures transparency and rigor [169].

### 2.1 Ethical Approval and Informed Consent

Ethical approval and informed consent were not applicable as this is a review article. However, the review adhered to ethical standards in selecting and analyzing the included studies.

## 3 Conclusion and Discussion

MGH is a promising and natural therapeutic option in medical treatments. It can enhance wound healing outcomes while significantly improving overall patient care. With its unique properties, MGH offers a natural therapeutic alternative for medical treatments. Honey contains phenolic compounds like phenolic acids and flavonoids, essential for wound healing. Their potent antioxidant properties protect tissues from oxidative damage and maintain vital biological processes. These compounds also have significant antibacterial properties that help to keep the wound environment sterile.

In addition, phenolics enhance angiogenesis, regulate inflammation, encourage cell division, and aid in tissue regeneration. Although the phenolic chemicals found in honey show promise in healing wounds, their exact molecular pathways are not yet clear. More research is required to fully understand their interactions, impact on cellular pathways, and possible synergistic effects. We recommend conducting new laboratory and clinical studies to determine whether other plant‐derived compounds, with structures and functions similar to those of phenolic compounds in honey, can perform as well as or better than honey in wound healing. These studies could significantly contribute to advancements in wound care.

The versatility and innate qualities of MGH render it a valuable adjunctive treatment for wound healing. Nevertheless, variations in honey's composition and potency, influenced by factors such as its floral source, present a challenge in achieving consistent quality and effectiveness in wound care. This challenge underscores the need for ongoing research to optimize its application in clinical settings.

Despite its promising mechanism, the clinical effectiveness of honey in accelerating wound healing remains a subject of debate. While some recent RCTs have reported positive outcomes, such as faster healing rates, reduced infection rates, and improved overall wound appearance, others have found no significant difference compared to standard care.

Future research should focus on medical‐grade honey's long‐term safety and efficacy, especially as its usage in chronic wound treatment grows. While honey has shown efficacy against antibiotic‐resistant bacteria, further research is needed to discover whether chronic use may lead to resistance to its antibacterial properties. Researchers should investigate whether honey may maintain its healing properties over time without causing fibrosis. Furthermore, it is critical to examine how long‐term honey consumption impacts patients' quality of life, considering factors such as ease of application, pain control, and wound odor reduction.

Researchers are encouraged to enhance MGH's antimicrobial activity and therapeutic properties by genetically modifying honey‐producing bees to accelerate wound healing. This strategy could make MGH a more competitive alternative to conventional treatments, potentially replacing other therapies with undesirable side effects.

Furthermore, the holistic benefits of honey‐based medications contribute to potential cost reduction and energy efficiency. With diverse medication options for different stages of wound healing, global availability, and lower energy consumption compared to pharmaceutical alternatives, honey‐based treatments offer a comprehensive solution. Moreover, their efficacy across various wounds, minimal side effects, and low reports of microbial resistance enhance their appeal, positioning honey‐based medications as promising contributors to cost‐effective healing solutions. To fully unlock its therapeutic potential, rigorous exploration is required to determine optimal concentrations, application modes, standardization protocols, and potential synergistic effects when combined with other treatment modalities

With further study and a deeper understanding of MGH's multiple benefits, it has the potential to revolutionize wound management, providing a natural and highly effective treatment option for patients worldwide.

## Author Contributions

Conceptualization: Ahmadreza Mehrbian, Parmis Barazesh, Helia Hajihassani. Data curation: Ahmadreza Mehrabian, Parmis Barazesh, Helia Hajihassani. Funding acquisition: none. Investigation: Parmis Barazesh, Helia Hajihassani, Fatemeh Motalebi, Seyedeh Mobina Hosseini Neiresi, Romina Hajihassani, Ahmadreza Mehrabian. Methodology: Ahmadreza Mehrbian, Parmis Barazesh, Helia Hajihassani. Project administration: Ahmadreza Mehrabian. Resources: Parmis Barazesh, Helia Hajihassani, Fatemeh Motalebi, Seyedeh Mobina Hosseini Neiresi, Romina Hajihassani, Ahmadreza Mehrabian. Software: Parmis Barazesh, Helia Hajihassani, Fatemeh Motalebi, Seyedeh Mobina Hosseini Neiresi, Romina Hajihassani, Ahmadreza Mehrabian Supervision: Ahmadreza Mehrabian. Validation: Ahmadreza Mehrbian, Parmis Barazesh, Helia Hajihassani. Visualization: Parmis Barazesh, Helia hajihassani. Writing–original draft: Ahmadreza Mehrabian, Parmis Barazesh, Helia Hajihassani, Fatemeh Motalebi, Seyedeh Mobina Hosseini Neiresi, Romina Hajihassani. Writing–review and editing: Ahmadreza Mehrabian, Parmis Barazesh, Helia Hajihassani.

## Conflicts of Interest

The authors declare no conflicts of interest.

## Data Availability

The data (NCBI) that support the findings will be available in PubChem.
